# Accuracy of a community mental health education and detection (CMED) tool for common mental disorders in KwaZulu-Natal, South Africa

**DOI:** 10.1186/s13033-022-00554-7

**Published:** 2022-08-23

**Authors:** Merridy Grant, Inge Petersen, Londiwe Mthethwa, Zamasomi Luvuno, Arvin Bhana

**Affiliations:** 1 Centre for Rural Health, School of Nursing and Public Health, University of KwaZulu-Natal, Durban, South Africa; 2 Health Systems Research Unit, South African Medical Research Council, Durban, South Africa

**Keywords:** Mental health, Community health workers, Screening, Low- and middle-income countries

## Abstract

**Background:**

Screening tools for mental health disorders improve detection at a primary health care (PHC) level. However, many people with mental health conditions do not seek care because of a lack of knowledge about mental health, stigma about mental illness and a lack of awareness of mental health services available at a PHC facility level. Interventions at a community level that raise awareness about mental health and improve detection of mental health conditions, are thus important in increasing demand and optimising the supply of available mental health services. This study sought to evaluate the accuracy of a Community Mental Health Education and Detection (CMED) Tool in identifying mental health conditions using pictorial vignettes.

**Methods:**

Community Health Workers (CHWs) administered the CMED tool to 198 participants on routine visits to households. Consenting family members provided basic biographical information prior to the administration of the tool. To determine the accuracy of the CMED in identifying individuals in households with possible mental health disorders, we compared the number of individuals identified using the CMED vignettes to the validated Brief Mental Health (BMH) screening tool.

**Results:**

The CMED performed at an acceptable level with an area under the curve (AUC) of 0.73 (95% CI 0.67–0.79), identifying 79% (sensitivity) of participants as having a possible mental health problem and 67% (specificity) of participants as not having a mental health problem. Overall, the CMED positively identified 55.2% of household members relative to 49.5% on the BMH.

**Conclusion:**

The CMED is acceptable as a mental health screening tool for use by CHWs at a household level.

## Introduction and background

Thirty percent of South Africans experience a common mental health condition in their lifetime [1] and only 7.5% of the uninsured population access care for these conditions [2]. To address this gap, the South African Department of Health has adopted the integration of mental health care into routine services, including screening for mental health disorders, at a primary health care (PHC) level using a task sharing approach as a policy imperative [3]. While this may increase service availability, a lack of perceived need or awareness of signs and symptoms and lack of awareness of service availability impacts demand for mental health services [4, 5]. Additionally, stigma and misinformation about mental health and treatment pose barriers to help seeking, particularly in African contexts where mental illness is highly stigmatised [6]. Integration of mental health services at a community level is fundamental to strengthening health systems [7] including interventions that increase mental health literacy to improve help seeking for mental health disorders [6, 8] and mental health screening to improve detection of mental health problems at a community level [5].

Screening tools for mental health have shown to improve detection of disorders at a community level and are an important first step along the treatment cascade to reduce the treatment gap [5, 8]. It is important that screening tools developed are culturally appropriate [9] particularly when used at a community level [10, 11]. Checklist based screening tools are mostly developed in high income contexts and often lack cultural sensitivity [9]. Detection at a community level by lay workers using an alternative prototype matching approach has been found to promote mental health service use in developing contexts [4, 5, 8]. Prototype matching involves detecting a mental health condition by matching a patient’s symptoms against a prototype paragraph-length description of the condition [12].

Community health workers (CHWs) provide an important link between communities and PHC facilities and have been found effective in detection and linkage to care for physical [13–16] and mental health conditions in low- and middle-income countries [LMICs] [5, 8]. The South African Department of Health (DoH) has implemented a PHC re-engineering strategy which includes the development of PHC community health teams formally known as Ward based PHC outreach teams with a focus on health promotion and prevention at community level [17]. The teams are made up of CHWs supervised by an Outreach Team Leader (OTL), usually an enrolled or professional nurse, and are linked to PHC facilities [18]. Their central role is health promotion, screening and linkage to care at a PHC facility level [19]. Up until now, they have mainly focused on physical health conditions.

This study is part of the Southern African Research Consortium for Mental health INTegration (SMhINT) project in collaboration with the KwaZulu-Natal Department of Health (KZN DoH), which has been using implementation science to evaluate the implementation of a collaborative care package for integrated primary mental health care (known as the Mental health INTegration [MhINT] package) for widespread scale-up using a learning health system approach [20]. The implementation strategies used in MhINT to implement and scale up the collaborative care package include training, supporting tools and materials, and the use of continuous quality improvement [CQI] [21, 22].

The need to strengthen identification of common mental disorders at a PHC level was identified through the first stage evaluation of the original MhINT package [23, 24]. This prompted the development and validation of the Brief Mental Health Screening Tool [BMH] [25] which has since been adopted by the KwaZulu-Natal DoH in the battery of screening tests offered at the PHC facility level as part of routine care. The BMH is a 7-item screening tool comprised of brief versions of the Alcohol Use Disorders Identification Test (AUDIT), the Patient Health Questionnaire (PHQ-9) and the General Anxiety Scale (GAD-7), and was validated for use within PHC facilities. In this regard the gold standard was professional nurse diagnosis using the Adult Primary Care tool which is a nationally adopted integrated set of chronic care guidelines used by PHC nurses in South Africa [26]. The mental health component in the Adult Primary Care manual is based on the WHO Mental Health Gap Action Programme Intervention Guide [27]. If screened positive on the BMH screening tool the patient is referred on for assessment to a professional nurse (fully registered nurse) who uses the Adult Primary Care manual as part of routine practice to assess and diagnose patients, offering brief psycho-education and onward referral for appropriate treatment.

A lack of a clear mental health pathway for care at a community level was also identified through the first stage evaluation of MhINT, and like the PHC context, no existing tools for identifying people with mental health problems were available in the community package of services that routinely include identification tools for physical health conditions such as TB, diabetes and HIV. Additionally, a need to expand detection of common mental disorders (CMDs) to a community level was identified by the DoH and a lack of a standardised screening tool for this purpose was noted. Initial consideration of the BMH tool was eschewed given that it focused on symptoms and commonly used psychiatric labels which was felt to be outside the scope of CHWs. Further, given low levels of mental health literacy in South African contexts [6], the DoH was concerned that this approach could result in labelling and stigmatisation of people who screened positive for a CMD in the community. The risk of this outcome is lessened at a PHC facility level where the BMH is administered by a nurse and confidentiality and patients’ rights are regulated by legal and professional ethical codes of conduct. The South African DoH and the KZN DoH thus requested the SMhINT team to develop and validate a mental health tool for use by CHWs to identify household members with possible mental health problems during their routine household visits. This gave rise to the development of the Community Mental Health Education and Detection (CMED) Tool [11] which was informed by the development of the Community Informant Detection Tool (CIDT) used to promote help-seeking among people with mental health disorders in Nepal as part of the Programme for Improving Mental Health Care (PRIME) [10]. Apart from helping to identify individuals at a household level who may have mental health problems, the CMED was extended to include psychoeducation about these mental health problems as well as elements of a healthy lifestyle to educate households on what can be done to improve one’s emotional health at home [11]. The addition of mental health education reduces the potential to promote labelling in contexts where mental health literacy is poor.

**Fig. 1:**
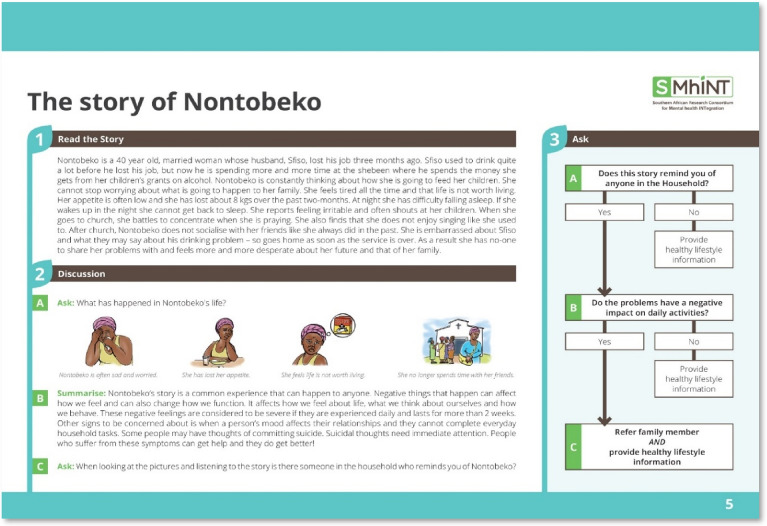
Vignette for depression (Nontobeko)

**Fig. 2:**
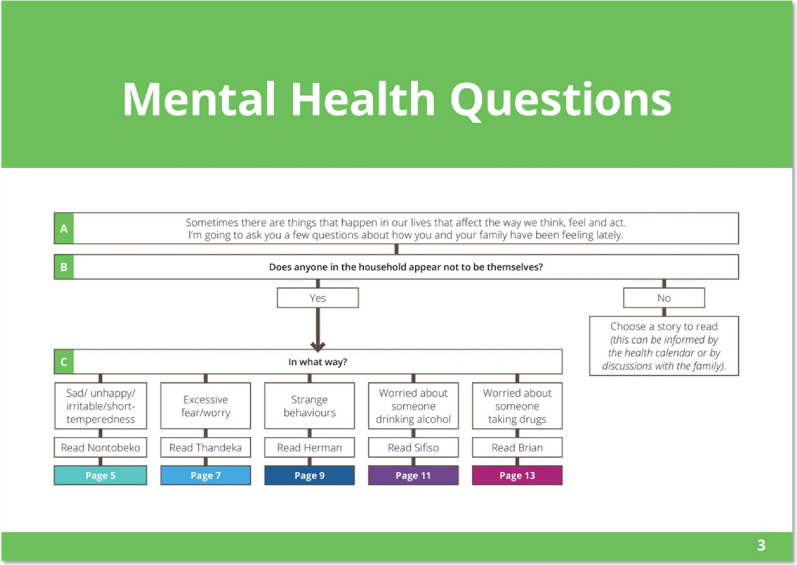
Mental health question flowchart

Using a prototype matching approach, the CMED incorporates local idioms in five separate case vignettes (prototype paragraphs) and related illustrations to facilitate identification of individuals with possible characteristics associated with depression, anxiety, psychosis, harmful alcohol and drug use [11]. The prototype paragraph avoids the use of labels and diagnostic categories using vignettes that describe functional impairment of some of the most common mental disorders in everyday language. Each vignette includes a story about a character and is labelled according to the protagonists’ name e.g. Nontobeko is the protagonist in the story about a person with depression symptoms, the vignette is labelled as ‘The story of Nontobeko’ (Fig. 1) and not by an associated mental health condition, e.g., depression to avoid labelling and possible stigmatisation [11]. Following reading of the vignette, an interactive psychoeducation component has been added to raise mental health awareness about each condition. This is followed by two structured questions that guide the CHW in matching symptoms with the prototype vignette and determines if the family member requires a referral for further care (Fig. 1). A flowchart assists the CHW in determining which vignette to read in the household (Fig. 2).

The aim of this study was to assess the accuracy of the newly developed Community Mental Health Education and Detection (CMED) Tool against the BMH which constituted the next level of screening in the system in identifying adult individuals with possible mental health problems in the household at a community level.

## Methods

### Setting

The accuracy study was conducted in the Newcastle sub-district of the Amajuba District of KwaZulu-Natal province of South Africa, as part of the larger SMhINT study. The Newcastle sub-district [population of 389,117] [28] is comprised of both urban and rural areas and is serviced by a district and provincial hospital with fourteen PHC facilities and, at the time of the study, five community health teams.

#### Establishing accuracy of the CMED

To establish the accuracy of the CMED relative to a “gold standard”, several factors were considered. First, the CMED would need to be comparable to recognised screening tools, locally and internationally. Second, the measure should be validated on the population of interest. Third, the validation tool should form part of routine screening for mental health by the DoH at a PHC level. As a consequence the BMH screening tool [25] which was validated in PHC facilities in the Amajuba district was considered the most appropriate tool as it was in use by the DoH as part of the screening cascade at the PHC level. While the BMH only focuses on common mental disorders (depression and anxiety symptoms and substance misuse characteristics), it was deemed to be suitable as the CMED broadly screens for mental health problems. Further, the BMH screening tool is used in first stage screening of all patients arriving at a PHC facility level before a more comprehensive assessment is done using the Adult Primary Care tool [against which the BMH screening tool was validated] [25].

### Procedure

#### Training

One community health team, including 3 OTLs and 17 CHWs, selected through the learning health systems approach adopted in MhINT and described in the development of the CMED tool [11], received a 4-day training workshop on the basic concepts of psychosocial and mental health disorders, self-care and on how to use the CMED tool. Confidentiality and encouraging but never imposing health seeking was covered in the training. OTLs and CHWs were encouraged to refer patients as per existing DoH guidelines. The development of the training material and the training itself was a collaboration between the Centre for Rural Health, UKZN and the South Africa-HIV Addiction Technology Transfer Centre Network for the KZN DoH.

Following the training, the community health team administered the CMED tool over three weeks in their communities, and the accuracy of the tool was assessed.

#### Field work procedures

A project Research Assistant, with a mental health background, accompanied the CHWs on all household visits. The CHW first introduced the research assistant to the family and explained the purpose of the visit. The research assistant then had a discussion with the family about the study and willing family members consented using written informed consent. The research assistant observed the administration of the CMED by the CHW and recorded the household member/s responses to the algorithmic questions relating to the vignettes. Immediately following the delivery of the CMED, the research assistant administered the validated BMH to the household members. The research assistant recorded whether the CMED identified the same household member as a positive [in need of referral] or negative case.

A positive score on the CMED results from positive responses to: (a) a family member identifying with a vignette (reminds them of self/others in the household), and (b) if this has a negative impact on daily activities. Based on the following cut-off scores established for the BMH subscales through the BMH validation study, a positive score was generated if any individual scored above the cut-offs of ≥ 4 on the AUD_C or ≥ 3 on the PHQ2 or ≥ 3 on the GAD2 subscales [25].

#### Sample

Using a prevalence rate of 17% based on a 12-month prevalence estimate established by a large-scale population-based study of common mental disorders (16.5%) in South Africa [1], with sensitivity set at 0.9, specificity at 0.85, and a confidence interval of 0.1, a sample of 203 participants is required.

All family members 18 years and older visited by CHWs in households as part of routine care over three weeks were invited to participate in the study. Any family member unable to give written consent was excluded. A total of 202 participants were sampled.

#### Data analysis

Sensitivity was prioritized over specificity as the intention was to identify any possible mental health disorder. Simple descriptive analysis was used to describe the sample characteristics. Analysis of the performance of the CMED against the BMH was done using STATA 15.1 (StataCorp LLC, Texas, USA) to calculate receiver operating characteristics (ROC) tables and graphs.

## Results

Of the 198 adult family members sampled, 79% were female with close to half of the participants (47%) falling into the 20–39 years age group. Some household visits comprised interviews with a single-family member while at other times, more than one family member was present. In both instances, interviews proceeded following a single or multiple consent process. Just over half (54.5%) of the CMED consultations took place with multiple family members and 45% were individual consultations (Table 1). Using the mental health flowchart (Fig. 2) CHW’s identified the story of Nontobeko (depression) to be relevant to 44% of households making it the story that was most frequently read in households, while the story of Brian (harmful drug abuse) was deemed relevant to only 5%.

**Table 1:** Sample characteristics (N = 198)

Characteristic	Gender				OVERALL	
Age	M	%	F	%	n	Percent
17–19 years	9	22.5	5	3.2	14	7.1
20–29 years	14	35	38	24.4	52	26.5
30–39 years	7	17.5	34	21.8	41	20.9
40–49 years	3	14.3	18	11.5	21	10.7
50–59 years	1	2.5	18	11.5	19	9.7
60–69 years	2	5	28	17.9	30	15.3
70+ years	4	10	15	9.6	19	9.7

	N	Percent
Gender		
Female	157	79.3
Male	41	20.7
Type of interview		
Individual	89	44.9
Family	108	54.5
Vignettes read in household		
Nontobeko (depression)	87	43.9
Sifiso (harmful alcohol use)	33	16.7
Herman (severe mental illness)	42	21.2
Thandeka (trauma related anxiety)	22	11.1
Brian (harmful drug use)	10	5.1

CHWs identified 106 participants as possible positive cases for a mental health disorder in need of referral to the PHC facility and 86 participants were considered possible negative cases, i.e., those without a possible mental health disorder (Table 2).

**Table 2:** CMED and BMH identified cases

CMED cases	Number of positive cases	Percent (N = 192)
Depression	47	24
Severe Mental Illness	25	13
Alcohol Abuse	19	10
Drug Use	9	4.7
Trauma related anxiety	6	3.1
Total CMED Positive	106	55.2
Total CMED Negative	86	44.8
Missing	8	

BMH Identified Cases		
BMH SCALE	Number of Positive Cases	Percent (N = 198)
AUD_C (≥ 4)	34	17.2
PHQ2 (≥ 3)	56	28.3
GAD2 (≥ 3)	60	30.3
BMH (Positive for ANY condition)	98	49.5
BMH (Negative for ANY condition)	99	50.3

Table 2 shows the specific problems identified using the CMED and the BMH. However, given that the CHWs function is to identify any mental health problem, only the overall positive or negative CMED scores were used in relation to the BMH reference standard. Cronbach alphas for the AUD-C was 0.87, 0.69 for the PHQ-2 and 0.65 for GAD-2.

**Fig. 3:**
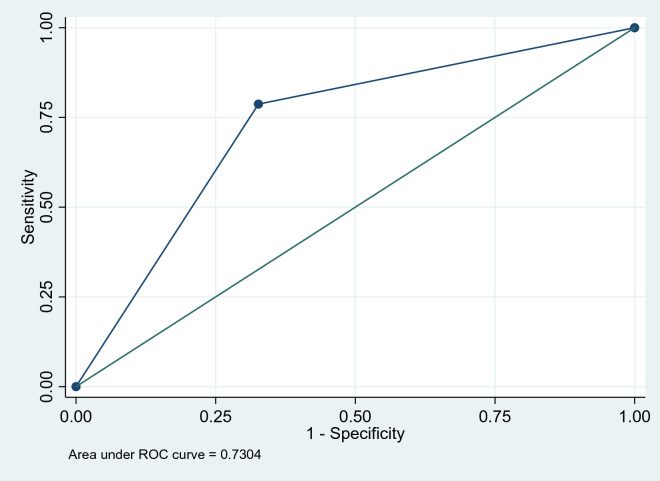
ROC curve for the CMED

An area under the curve (AUC) ≥ 0.70 is considered fair/acceptable, ≥ 0.80 is good/excellent and ≥ 0.90 excellent/outstanding [29, 30]. ROC survey analysis shows that the CMED had an acceptable AUC of 0.73 (95% CI 0.67–0.79) (Fig. 3). It accurately identified 79% (sensitivity) of participants as having a possible mental health disorder and accurately identified 67% (specificity) of participants as not having a mental health disorder with a 73% probability (Table 3). The associated Positive Predictive Value (PPV) and Negative Predictive Value (NPV) (the proportions of positive and negative results that are true positive and true negative results, respectively) were 70% and 77%.

**Table 3:** Performance of the CMED in identifying mental health problems at a household level (N = 192)

Cut-point	Sensitivity (%)	Specificity (%)	Correctly classified (%)	LR +	LR−
≥ 0	100	0	48.96	1.00	0.00
≥ 1	78.72	67.35	72.92	2.41	0.32
> 1	0	100	51.04	0.00	1.000

## Discussion

The CMED was assessed to have acceptable accuracy in identifying people with possible mental health disorders in the community. With a sensitivity of 79%, the tool has the potential for early identification and referral for further assessment and treatment among individuals who might otherwise not access any mental health services. A specificity of 67% is a relatively good result and suggests the PHC facilities will not be over-burdened with false positive cases.

This finding is similar to previous studies that demonstrate that community mental health detection using a prototype matching approach can assist in identifying community members with possible mental health symptoms in need of onward referral with acceptable accuracy [4, 31]. With 79% sensitivity, the CMED demonstrates that it can accurately detect individuals in the community with mental health problems 73% of the time. The CMED has a positive predictive value of 0.70 (i.e., the proportion of positive test results that are true positives) which is very similar to that obtained in the community informant detection tool (CIDT) used in Nepal [0.68] [4]. This is a remarkable, especially since the CHWs in our study have had no previous formal training or education around mental health. The CMED thus shows promise as an approach to mental health case detection at a community level using non-stigmatising characterizations.

In the context where 30% of South Africans experience a common mental health condition in their lifetime [1], and where only 7.5% of the uninsured population access care for these conditions [2], the need to increase supply and demand for mental health services is highlighted. Low mental health literacy, lack of knowledge about service availability, stigma and misinformation about treatment pose barriers to help seeking. In the context of low levels of mental health literacy in African contexts [6], interventions at a community level, such as the CMED tool, are important to improve communities understanding of mental health problems, community identification and awareness of service availability and help seeking [10]. An added benefit of the CMED tool is that it is context sensitive and uses local idioms and illustrations to encourage conversations about mental health between CHWs and families in a non-stigmatising manner [11] during routine home visits, whilst simultaneously assisting with screening and referral for care.

The CMED tool may also be useful in tracing visits to link loss-to-follow-up patients to care, with the tool being helpful in identifying mental health problems that may be driving non-adherence. This is of particular importance as mental disorders have a mutually reinforcing relationship with non-communicable diseases, compromising both prevention and treatment through exacerbating modifiable risk factors and compromising adherence and self-care [32].

## Study limitations

Limitations of this study include that the study was limited to one community health team in a sub-district in one region of the country. The CMED was administered in a household setting and some participants could have been reluctant to openly share their mental health experiences in the presence of other family members, particularly regarding harmful alcohol and drug use. The BMH, on the other hand, was administered individually in private after the CHW consult and this could account for the difference in some CMED and BMH scores. The CHWs lack of familiarity with the tool and the presence of researchers observing the administration of the tool (for both CHWs and household members) could also have influenced the findings.

## Conclusion

The CMED has acceptable accuracy for facilitating recognition of mental health problems at the community level. Further research is required to establish the acceptability of the tool by community health teams and households as well as how best to integrate the use of the tool as part of routine screening for other conditions. While the CMED includes providing psychoeducation to improve mental health literacy, there is a need to demonstrate the CMEDs utility in improving mental health literacy, knowledge of and uptake of mental health services and reducing stigma as it becomes part of CHWs routine visits.

## Data Availability

The datasets generated and/or analysed during the current study will be available in the National Institute of Mental Health Data Archive (NDA) as per the data sharing agreement.

## Abbreviations

AUDIT: Alcohol use disorders identification test
AUC: Area under the curve
BMH: Brief mental health screening tool
BREC: Biomedical Research Ethics Committee
CMED: Community Mental Health Education and Detection Tool
CMD: Common mental disorder
CHW: Community health worker
CIDT: Community informant detection tool
CQI: Continuous quality improvement
GAD: General anxiety scale
KZN DoH: KwaZulu-Natal Department of Health
MhINT: Mental health INTegration package
NPV: Negative predictive value
OTL: Outreach team leader
PHQ: Patient health questionnaire
PHC: Primary health care
PPV: Positive predictive value
PRIME: Programme for improving mental health care
ROC: Receiver operating characteristics
SA DoH: South African Department of Health
SMhINT: Southern African Research Consortium for Mental health INTegration project

## References

[1] Herman AA, Stein DJ, Seedat S, Heeringa SG, Moomal H, Williams DR (2009). The South African Stress and Health (SASH) study: 12-month and lifetime prevalence of common mental disorders. S Afr Med J.

[2] Docrat S, Besada D, Cleary S, Daviaud E, Lund C (2019). Mental health system costs, resources and constraints in South Africa: a national survey. Health Policy Plan.

[3] South African National Department of Health. National mental health policy framework and strategic plan 2013–2020. Pretoria NDoH; 2013.

[4] Jordans MJ, Kohrt BA, Luitel NP, Komproe IH, Lund C (2015). Accuracy of proactive case finding for mental disorders by community informants in Nepal. Br J Psychiatry.

[5] Jordans MJ, Luitel NP, Lund C, Kohrt BA (2020). Evaluation of proactive community case detection to increase help seeking for mental health care: a pragmatic randomized controlled trial. Psychiatr Serv.

[6] Egbe CO, Brooke-Sumner C, Kathree T, Selohilwe O, Thornicroft G, Petersen I (2014). Psychiatric stigma and discrimination in South Africa: perspectives from key stakeholders. BMC Psychiatry.

[7] Sheikh K, George A, Gilson L (2014). People-centred science: strengthening the practice of health policy and systems research. Health Res Policy Syst.

[8] Shidhaye R, Murhar V, Gangale S, Aldridge L, Shastri R, Parikh R (2017). The effect of VISHRAM, a grass-roots community-based mental health programme, on the treatment gap for depression in rural communities in India: a population-based study. Lancet Psychiatry.

[9] Bass JK, Bolton PA, Murray LK (2007). Do not forget culture when studying mental health. Lancet.

[10] Subba P, Luitel NP, Kohrt BA, Jordans MJD (2017). Improving detection of mental health problems in community settings in Nepal: development and pilot testing of the community informant detection tool. Confl Heal.

[11] Grant M, Luvuno Z, Bhana A, Mntambo N, Gigaba S, Ntswe E (2021). The development of a Community Mental Health Education and Detection (CMED) Tool in South Africa. Soc Sci Med Mental Health.

[12] Westen D (2012). Prototype diagnosis of psychiatric syndromes. World Psychiatry.

[13] Bhutta Z, Lassi Z, Pariyo G, Huicho L. Global experience of community health workers for delivery of health related millennium development goals: a systematic review, country case studies, and recommendations for integration into National Health Systems. WHOGlobal Health Workforce Alliance (GHWA)Geneva: World Health Organization. Global Health Workforce Alliance World Health Organization. 2010;1.

[14] Zulu JM, Kinsman J, Michelo C, Hurtig AK (2014). Integrating national community-based health worker programmes into health systems: a systematic review identifying lessons learned from low-and middle-income countries. BMC Public Health.

[15] Naidoo N, Railton J, Jobson G, Matlakala N, Marincowitz G, McIntyre JA (2018). Making ward-based outreach teams an effective component of human immunodeficiency virus programmes in South Africa. S Afr J HIV Med.

[16] Horwood C, Butler L, Barker P, Phakathi S, Haskins L, Grant M (2017). A continuous quality improvement intervention to improve the effectiveness of community health workers providing care to mothers and children: a cluster randomised controlled trial in South Africa. Hum Resour Health.

[17] South African National Department of Health (2010). Re-engineering primary health care in South Africa.

[18] South African National Department of Health (2017). Policy framework and strategy for ward based primary health care outreach teams.

[19] Schneider H, Besada D, Sanders S, Daviaud E, Rhode S (2018). Ward-based primary health care outreach teams in South Africa developments, challenges and future directions. South African Health Review 2018.

[20] Petersen I, Kemp CG, Rao D, Wagenaar BH, Sherr K, Grant M (2021). Implementation and scale-up of integrated depression care in South Africa: an observational implementation research protocol. Psychiatr Serv.

[21] Institute for Healthcare Improvement. The breakthrough series: IHI’s collaborative model for achieving breakthrough improvement. Boston; 2003.

[22] O'Neill SM, Hempel S, Lim YW, Danz MS, Foy R, Suttorp MJ (2011). Identifying continuous quality improvement publications: what makes an improvement intervention ‘CQI’?. BMJ Qual Saf.

[23] Kemp CG, Mntambo N, Bachmann M, Bhana A, Rao D, Grant M (2020). Patient-level predictors of detection of depressive symptoms, referral, and uptake of depression counseling among chronic care patients in KwaZulu-Natal, South Africa. Global Mental Health.

[24] Kemp CG, Mntambo N, Weiner BJ, Grant M, Rao D, Bhana A (2021). Pushing the bench: a mixed methods study of barriers to and facilitators of identification and referral into depression care by professional nurses in KwaZulu-Natal. S Afr SSM Mental Health.

[25] Bhana A, Mntambo N, Gigaba SG, Luvuno ZPB, Grant M, Ackerman D (2019). Validation of a brief mental health screening tool for common mental disorders in primary healthcare. S Afr Med J.

[26] Fairall L, Cornick R, Bateman E (2018). Empowering frontline providers to deliver universal primary healthcare using the practical approach to care kit. BMJ.

[27] World Health Organisation (2016). mhGAP intervention guide for mental, neurological and substance use disorders in non-specialized health settings V 2.

[28] Statistics South Africa. Provincial profile: KwaZulu-Natal. Community survey 2016. Pretoria; 2016.

[29] Mandrekar JN (2010). Receiver operating characteristic curve in diagnostic test assessment. J Thorac Oncol.

[30] Safari S, Baratloo A, Elfil M, Negida A (2016). Evidence based emergency medicine; part 5 receiver operating curve and area under the curve. Emerg.

[31] Mohsin S, Waqas A, Atif N, Rabbani MW, Ali Khan S, Bilal S (2021). Accuracy of community informant led detection of maternal depression in rural Pakistan. Int J Environ Res Public Health.

[32] Prince M, Patel V, Saxena S, Maj M, Maselko J, Phillips MR (2007). No health without mental health. Lancet.

